# DeepGO: predicting protein functions from sequence and interactions using a deep ontology-aware classifier

**DOI:** 10.1093/bioinformatics/btx624

**Published:** 2017-10-03

**Authors:** Maxat Kulmanov, Mohammed Asif Khan, Robert Hoehndorf

**Affiliations:** Computer, Electrical and Mathematical Sciences & Engineering Division, Computational Bioscience Research Center, King Abdullah University of Science and Technology, Thuwal, Kingdom of Saudi Arabia

## Abstract

**Motivation:**

A large number of protein sequences are becoming available through the application of novel high-throughput sequencing technologies. Experimental functional characterization of these proteins is time-consuming and expensive, and is often only done rigorously for few selected model organisms. Computational function prediction approaches have been suggested to fill this gap. The functions of proteins are classified using the Gene Ontology (GO), which contains over 40 000 classes. Additionally, proteins have multiple functions, making function prediction a large-scale, multi-class, multi-label problem.

**Results:**

We have developed a novel method to predict protein function from sequence. We use deep learning to learn features from protein sequences as well as a cross-species protein–protein interaction network. Our approach specifically outputs information in the structure of the GO and utilizes the dependencies between GO classes as background information to construct a deep learning model. We evaluate our method using the standards established by the Computational Assessment of Function Annotation (CAFA) and demonstrate a significant improvement over baseline methods such as BLAST, in particular for predicting cellular locations.

**Availability and implementation:**

Web server: http://deepgo.bio2vec.net, Source code: https://github.com/bio-ontology-research-group/deepgo

**Supplementary information:**

Supplementary data are available at *Bioinformatics* online.

## 1 Introduction

Advances in sequencing technology have led to a large and rapidly increasing amount of genetic and protein sequences, and the amount is expected to increase further through sequencing of additional organisms as well as metagenomics. Although knowledge of protein sequences is useful for many applications, such as phylogenetics and evolutionary biology, understanding the behavior of biological systems additionally requires knowledge of the proteins’ functions. Identifying protein functions is challenging and commonly requires *in vitro* or *in vivo* experiments (Costanzo *et al.*, 2016), and it is obvious that experimental functional annotation of proteins will not scale with the amount of novel protein sequences becoming available.

One approach to address the challenge of identifying proteins’ functions is the computational prediction of protein functions (Radivojac *et al.*, 2013). Function prediction can use several sources of information, including protein–protein interactions (Hou, 2017; Jiang and McQuay, 2012; Kirac and Ozsoyoglu, 2008; Nguyen *et al.*, 2011; Sharan *et al.*, 2007), genetic interactions (Costanzo *et al.*, 2016), evolutionary relations (Gaudet *et al.*, 2011), protein structures and structure prediction methods (Konc *et al.*, 2013), literature (Verspoor, 2014) or combinations of these (Sokolov and Ben-Hur, 2010). These methods have been developed for many years, and their predictive performance is improving steadily (Radivojac *et al.*, 2013).

There are several key challenges for protein function prediction methods. One of these is the complex relation between protein sequence, structure and function (Alberts *et al.*, 2002); despite significant progress in the past years in protein structure prediction (Moult *et al.*, 2014), it still requires large efforts to predict protein structure with sufficient quality to be useful in function prediction. Another challenge is the large and complex output space for any classification method. Protein functions are classified using the Gene Ontology (GO) (Ashburner *et al.*, 2000) which contains over 40 000 functions and cellular locations. Additionally, the GO contains strong, formally defined relations between functions that need to be taken into account during function prediction to ensure that these predictions are consistent (Radivojac *et al.*, 2013; Sokolov and Ben-Hur, 2010). The formal dependencies between classes in GO also lead to the situation where proteins are assigned to multiple function classes in GO, for different levels of abstraction. Furthermore, several proteins do not only have a single function but may be peiotropic and have multiple different functions, making function prediction inherently a multi-label, multi-class problem. A final challenge is that proteins do not function in isolation. In particular higher-level physiological functions that go beyond simple molecular interactions, such as *apoptosis* or *regulation of heart rate*, will require other proteins and cannot usually be predicted by considering a single protein in isolation. Due to these challenges, it is also not obvious what kind of features should be used to predict the functions of a protein, and whether they can be generated efficiently for a large number of proteins.

Here, we present a novel method for predicting protein functions from protein sequence and known interactions. We combine two forms of representation learning based on multiple layers of neural networks to learn features that are useful for predicting protein functions, one method that learns features from protein sequence and another that learns representations of proteins based on their location in an interaction network. We then utilize these features in a novel deep neuro-symbolic model that is built to resemble the structure and dependencies between classes that exist within the GO, refine predictions and features on each level of GO, and ultimately optimize the performance of function prediction based on the performance over the whole ontology hierarchy.

We demonstrate that our model improves performance of function prediction over a BLAST baseline, and performs particularly well in predicting cellular locations of proteins. The main advantage of our approach is that it does not rely on manually crafted features but is entirely data-driven.

## 2 Materials and methods

### 2.1 Datasets

For our experiments, we use the Gene Ontology (GO) (Ashburner *et al.*, 2000), downloaded on 05 January 2016 from http://geneontology.org/page/download-ontology in OBO format. The version of GO has 44 683 classes of which 1968 are obsolete. GO has three major branches, one for biological processes (BP), molecular functions (MF) and cellular components (CC), each containing 28 647, 10 161 and 3907 classes, respectively.

We use SwissProt’s (Boutet *et al.*, 2016) reviewed and manually annotated protein sequences with GO annotations downloaded on 05 January 2016 from http://www.uniprot.org/uniprot/. The dataset contains 553 232 proteins, and 525 931 proteins have function annotations. Furthermore, we select proteins with annotations with experimental evidence code (EXP, IDA, IPI, IMP, IGI, IEP, TAS and IC) and filter the proteins by maximum length of 1002 ignoring proteins with ambiguous amino acid codes (B, O, J, U, X, Z) in their sequence. Our final dataset contains 60 710 proteins annotated with 27 760 classes (19 181 in BP, 6221 in MF and 2358 in CC). The dataset covers more than 90% of all proteins with experimental annotations in SwissProt. Supplementary Figure S1 shows the sequence length distribution.

### 2.2 Training

We trained three models, one for each sub-ontology in GO. First, we propagate annotations using the GO ontology structure and randomly split proteins into a training set (80%) and testing set (20%). Due to computational limitations and the small number of annotations for very specific GO classes, we ranked GO classes by their number of annotations and selected the top 932 terms for BP, 589 terms for MF and 436 terms for the CC ontology. These cutoff values correspond to selecting only classes with the minimum number of annotations 250, 50 and 50, for BP, MF and CC, respectively.

We create three binary label vectors for each protein sequence, one for each of the GO hierarchies. If a protein sequence is annotated with a GO class from our lists of selected classes, then we assign 1 to the term’s position in the binary label vector and use it as positive sample for this term. Otherwise, we assign 0 and use it as negative sample. For training and testing, we use proteins which have been annotated with at least one GO term from the set of the GO terms for the model.

### 2.3 Data representation

The input of our model is the amino acid (AA) sequence of a protein. Each protein is a character sequence composed of 20 unique AA codes. We generate trigrams of AA from the protein sequence. The trigrams can be represented as one-hot encoding vectors of length 8000; however, the sparse nature of one-hot encodings only provides a limited generalization performance. To address this limitation, we use the notion of dense embeddings (Bengio *et al.*, 2003; Hinton, 1986). An embedding is a lookup table used for mapping each code in a vocabulary to a dense vector. Initially, we initialized the vectors randomly and then learn the actual vector-based representations as an additional layer in our network architecture during training. This approach allows us to learn meaningful vectors, i.e. vectors that resemble correlations within the data that can be utilized as features to predict protein functions. We have also performed experiments (on a smaller dataset) with one-hot encodings of AA trigrams, and found that dense representation performs better than one-hot encoding.

We built a vocabulary of unique AA trigrams where each trigram is represented by its 1-based index. Using this vocabulary, we encoded a sequence of length 1002 as a vector of 1000 indices. If the length of the sequence is less than 1002, we pad the vector with zeros. We ignore all the proteins with sequence length more than 1002. The first layer in the deep learning model is intended to learn embeddings where each index is mapped to a dense vector by referring to a lookup table, using an embedding size of 128 and therefore representing a protein sequence of length of 1002 as a matrix of 1000 × 128.

### 2.4 Convolutional neural network

Convolutional Neural Networks (CNNs) are biologically inspired NN which try to mimic the receptive field of biological neuron. In CNNs, convolution operations are applied over the input layer to compute the output (LeCun and Bengio, 1998). They exploit local correlation by enforcing local connections between neurons of adjacent layers, where each region of the input is connected to a neuron in the output. Having multiple convolution filters helps in learning multiple features and providing insights into multiple facets of the data. In our work, we used 1-dimensional (1D) convolution over protein sequence data. The 1D convolution exploits sequential correlation. If we have an input g(x)∈[1,l]→ℝ and a kernel function f(x)∈[1,k]→ℝ, the convolution *h*(*y*) between *f*(*x*) and *g*(*x*) with stride *d* is defined as:

(1) $$h(y)=\sum_{x=1}^{k}f(x)\cdot g(y\cdot d-x+c)$$

where c=k−d+1 is an offset constant. The output *h_j_*(*y*) is obtained by a sum over *i* of the convolutions between *g_i_*(*x*) and *f_ij_*(*x*). The output vector *h* represents the feature map learned through convolution.

The resulting feature map will contain redundant information and is of significant size. Therefore, to reduce the feature space, redundant information is discarded through temporal max-pooling (Collobert *et al.*, 2011). This operation selects the maximum value over a window of some length *w*. The features after convolution and the temporal pooling layer are intended to be higher level representation of protein sequences which can then be used as input to fully connected layers for classification.

For our experiments, we used one 1D Convolution layer with 32 filters of size 128 which are applied on the embedding matrix of each sequence, and a 1D max-pooling layer with pool length of 64 and stride of 32. Each filter is intended to learn a specific type of feature, and multiple filters may enable learning of different aspects of the underlying data. The output of the 1D max-pooling layer is a vector with length of 832.

### 2.5 Protein–protein interaction (PPI) network features

In addition to protein sequences, we use protein–protein interaction (PPI) networks for multiple species from the STRING database (Szklarczyk *et al.*, 2015), filtered by confidence score of 300 and connected with orthology relations from the EggNOG database (Huerta-Cepas *et al.*, 2016) by creating a symmetric *ortholog-of* edge for each orthology group. To further separate proteins by the orthology group to which they belong, we introduce a new orthology relation for each orthology group in eggNOG. In total, the network consists of 8 478 935 proteins, 190 649 edge types and 11 586 695 610 edges. Using this heterogeneous network, we generated knowledge graph embeddings of size 256 for each protein (Alshahrani *et al.*, 2017).

Since our model is based on UniProt protein identifiers, we mapped nodes in the network to UniProt identifiers using the identifier mapping provided by STRING. We mapped 6 960 395 proteins in UniProt to our network and the resulting knowledge graph embeddings. For the proteins with missing network representations, we assigned a vector of zeros. We combined the knowledge graph embeddings for the nodes with the output of the max-pooling layer of length 832 as a combined feature vector.

### 2.6 Hierarchical classification layout

**Fig. 1.**
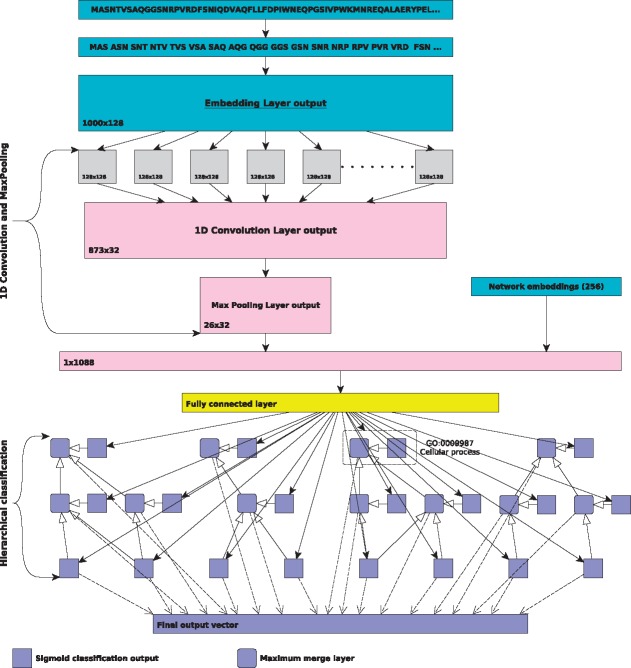
Convolutional Neural Network Architecture. (1) The input of the model is a list of integer indexes of trig.rams generated from protein sequence and vector of size 256 for protein PPI network representation. The trigram indexes are passed to an embedding layer which provides vector representations of size 128 for each trigram. The output of an embedding layer is a matrix of size 1000 × 128 on which we apply convolution and max-pooling. We merge the flattened output of the max-pooling layer and concatenate the resulting vector with the PPI network embeddings. This feature vector is then passed to hierarchically structured classification layers. (2) The hierarchically structured classification layers form a directed acyclic graph following the taxonomic structure of GO for is-a relations. For each GO class we generate one fully connected layer with a sigmoid activation function that predicts whether the input should be classified with this GO class. To ensure consistency, all non-leaf nodes in the graph use a maximum merge layers (rounded purple square) which outputs the maximum value of the classification results for all child nodes and the internal node’s classification results. The output vector of the model is the concatenation of maximum merge layers of the internal nodes and the classification layers of the leaf nodes

Using a fully connected layers for each class in GO, we created a hierarchical classification neural network model that encodes for transitivity of subclass relations. We use only the subclass relations and create a small neural network for each class in our subset of selected terms. The concatenated sequence and PPI network features are passed to a fully connected layer with 1024 neurons and its output is passed to the hierarchically structured neural networks for classification. Each network consists of one fully connected layer with a sigmoid activation function, and takes as an input the output of first fully connected layer. This layer is responsible for classifying the proteins for its term. To ensure consistent hierarchical classification, for each class which has children in GO, we created a merge layer which selects the maximum value of the classification layers of the term and its children. Finally, the output of the model is the concatenation of classification layers of leaf nodes and the maximum layers of internal nodes. Figure 1 shows the architecture of our neural network model.

### 2.7 Model implementation and optimization

In training, we minimize the multi-output binary cross entropy loss function using the Rmsprop optimizer (Tieleman and Hinton, 2012) with a mini batch of size 128 and learning rate of 0.01. Initially, the weights of our model are initialized according to a uniform distribution (Glorot and Bengio, 2010). We fit our model with 80% of our training set and use the remaining 20% of the training set as a validation set. At the end of each training epoch, we monitor the convergence of the model on the validation set and keep the weights of the best performing model. To prevent over-fitting of the model, we use dropout layers as regularizers. We implement our model using the deep learning library Keras with TensorFlow (Abadi *et al.*, 2016) as a backend. To accelerate the training process, we use NVIDIA Pascal X GPUs. The training time for the Biological Process ontology model (which is the largest model) is less than three hours and the inference time is less than one second. We manually tuned the following set of parameters: minibatch size, number of convolution filters, filter size, number of neurons in fully connected layer and learning rate. We select the best parameters depending on the value of validation loss. Supplementary Table S1 shows the validation losses for different embedding sizes and number of convolution filters. We observe only small differences in validation loss (based on binary cross entropy) for the different combinations of parameters we evaluate. Source code for our implementation is available at https://github.com/bio-ontology-research-group/deepgo.

### 2.8 BLAST baseline and comparison

We use the BLAST (Altschul *et al.*, 1997) sequence alignment method as a baseline to compare our model’s performance. We use BLAST to find the most similar sequence in a database of experimentally annotated proteins for a query sequence and assign all its annotations to the query sequence. We create a database for each ontology with a proteins in our training set that have been annotated with at least one term from the ontology. For a proteins in our test set, we use the BLASTP program to obtain the protein with the highest alignment score from our training set and assign all its functional terms to the protein from our test set.

For comparison, we obtain FFPred3 (Cozzetto *et al.*, 2016) prediction results for CAFA3 targets from http://bioinfadmin.cs.ucl.ac.uk/downloads/ffpred/cafa3/ and GoFDR (Gong *et al.*, 2016) results through the web service available at http://gofdr.tianlab.cn/. We apply these on a set of protein targets released on 05 June 2017 that had no function annotations at the time of training. The dataset contains 1367 proteins and 3619 annotations. It is available for download at https://github.com/bio-ontology-research-group/deepgo.

### 2.9 Evaluation

We evaluate our model performance with two measures (Clark and Radivojac, 2013) that are used in CAFA challenge (Radivojac *et al.*, 2013). The first measure is a protein centric maximum F-measure. Here, we compute F-measure for a threshold t∈[0,1] using the average precision for proteins for which we predict at least one term and average recall for all proteins. Then, we select the maximum F-measure of all thresholds. We compute the *F_max_* measure using the following formulas:

(2) $$pr_{i}(t)=\frac{\sum {}_{f}I(f\in P_{i}(t)\wedge f\in T_{i})}{\sum {}_{f}I(f\in P_{i}(t))}$$

(3) $$rc_{i}(t)=\frac{\sum {}_{f}I(f\in P_{i}(t)\wedge f\in T_{i})}{\sum {}_{f}I(f\in T_{i})}$$

(4) $$AvgPr(t)=\frac{1}{m(t)}\cdot \sum_{i=1}^{m(t)}pr_{i}(t)$$

(5) $$AvgRc(t)=\frac{1}{n}\cdot \sum_{i=1}^{n}rc_{i}(t)$$

(6) $$F_{\text{max}}=\underset{{}_{t}}{\max}\left\{\frac{2\cdot AvgPr(t)\cdot AvgRc(t)}{AvgPr(t)+AvgRc(t)}\right\}$$

In these measures, *f* is GO class, *P_i_*(*t*) is a set of predicted classes for a protein *i* using a threshold *t*, and *T_i_* is a set of annotated classes for a protein *i*. Precision is averaged over the proteins where we at least predict one term and *m*(*t*) is the total number of such proteins. *n* is a number of all proteins in a test set.

The second measure is a term-centric where for each term *f* we compute AUC of a ROC Curve of a sensitivity (or a recall) for a given false positive rate (1 - specificity). We compute sensitivity and specificity using the following formulas:

(7) $$sn_{f}(t)=\frac{\sum {}_{i}I(f\in P_{i}(t)\wedge f\in T_{i})}{\sum {}_{i}I(f\in T_{i})}$$

(8) $$sp_{f}(t)=\frac{\sum {}_{i}I(f\notin P_{i}(t)\wedge f\notin T_{i})}{\sum {}_{i}I(f\notin T)}$$

Here, *P_i_*(*t*) is a set of predicted terms for a protein *i* using a threshold *t* and *T_i_* is a set of annotated terms for a protein *i*. Additionally, we report a term-centric *F_max_* measure where for each term *f* we compute the F-measure using threshold *t* and all proteins in our test set. Then, we take the maximum for all the thresholds.

(9) $$pr_{f}(t)=\frac{\sum {}_{i}I(f\in P_{i}(t)\wedge f\in T_{i})}{\sum {}_{i}I(f\in P_{i}(t))}$$

(10) $$rc_{f}(t)=\frac{\sum {}_{i}I(f\in P_{i}(t)\wedge f\in T_{i})}{\sum {}_{i}I(f\in T_{i})}$$

(11) $$F_{\text{max}f}=\underset{{}_{t}}{\max}\left\{\frac{2\cdot pr_{f}(t)\cdot rc_{f}(t)}{pr_{f}(t)+rc_{f}(t)}\right\}$$

Additionally, we compute global ROC AUC for all predictions scores given by the models and Mathews Correlation Coefficient (MCC) for a threshold which gives a maximum protein centric F-measure. The ROC AUC is computed using the following formulas for a threshold parameter *t*:

(12) $$AUC=\int_{-\infty }^{\infty }TPR(t)(-FPR\prime (t))dt$$

(13) $$TPR(t)=\frac{TP(t)}{TP(t)+FN(t)},FPR(t)=\frac{FP(t)}{FP(t)+TN(t)}$$

TP is a number of true positives, FN is a number of false negatives, FP is a number of false positives and TN is a number of true negatives. The MCC is computed using the following formula:

(14) $$MCC=\frac{TP\cdot TN-FP\cdot FN}{\sqrt{\left(TP+FP)(TP+FN)(TN+FP)(TN+FN\right)}}$$

## 3 Results

### 3.1 Feature learning and neuro-symbolic hierarchical classification

We build a machine learning model that aims to address three challenges in computational function prediction: learning features to represent a protein, predicting functions in a hierarchical output space with strong dependencies, and combining information from protein sequences with protein–protein interaction networks. The first part of our model learns a vector representation for a protein sequence which can be used as features to predict protein functions. The second part of the model aims to encode for the functional dependencies between classes in GO and optimizes classification accuracy over the hierarchical structure of GO at once instead of optimizing one model locally for each class. The intention is that this model can identify both explicit dependencies between classes in GO, as expressed by relations between classes encoded in the ontology, as well as implicit dependencies such as frequently co-occurring classes. While a single model over the entire GO would likely yield best results, due to the size of the GO, we independently train three models for each of GO’s three sub-ontologies, Molecular Function (MF), Biological Process (BP) and Cellular Component (CC), and focus exclusively on subclass relations between GO classes. We generate a series of fully connected layers, one for each class *C* in the GO. Each of these layers has exactly one connection to an output neuron, *Out*(*C*), and, for each direct subclass *D* of *C*, a connection to another layer representing *D*. This architecture resembles the hierarchical structure of GO and the dependencies between its classes, ensures that discriminating features of each class can be learned hierarchically while taking into account the symbolic relations in GO. More generally, each dense layer of this ontology-structured neural network layout is intended to learn features that can discriminate between its subclasses. Figure 1 illustrates the basic architecture of our model.

We train three model in a supervised way (one model for each of the GO ontologies). For this purpose, we first split all proteins with manually curated GO annotations in SwissProt in a training set (80%) and an evaluation set (20%). We use the manually assigned GO functions of the proteins in the training set to train our models. The performance of each model is globally optimized over all the GO functions (within either the MF, BP, or CC hierarchy) through back-propagation. We then evaluate the performance of our model on the 20% of proteins not used for training, using the evaluation metrics developed and employed in the CAFA challenge (Radivojac *et al.*, 2013). Table 1 shows the overall performance of our model and the comparison to using BLAST to assign functions. We find that our model, which relies only on protein sequences (DeepGOSeq), outperforms BLAST in predicting cellular locations, but does not achieve improved performance compared to BLAST in the MF and BP ontologies when evaluated either on the full set of GO functions or the subset used by our model.

**Table 1.** Overview of our model’s performance and comparison to BLAST baseline

Method	BP					MF					CC				
	F _max_	AvgPr	AvgRc	AUC	MCC	F_max_	AvgPr	AvgRc	AUC	MCC	F_max_	AvgPr	AvgRc	AUC	MCC
BLAST	0.314	0.302	0.327			0.372	0.367	0.377			0.362	0.321	0.417		
DeepGOSeq	0.293	0.304	0.282	0.814	0.266	0.364	0.453	0.304	0.875	0.328	0.568	0.602	0.538	0.924	0.520
DeepGOFlat	0.387	0.393	0.382	0.899	0.395	0.451	0.529	0.393	0.925	0.428	0.632	0.635	0.629	0.966	0.595
DeepGO	**0.395**	0.412	0.379	0.896	0.397	**0.470**	0.577	0.397	0.928	0.438	**0.633**	0.643	0.624	0.967	0.592
BLAST (selected)	0.344	0.376	0.317			**0.541**	0.615	0.483			0.497	0.506	0.489		
DeepGOSeq (selected)	0.322	0.319	0.324	0.814	0.266	0.392	0.453	0.346	0.875	0.328	0.574	0.602	0.548	0.924	0.520
DeepGOFlat (selected)	0.425	0.415	0.436	0.899	0.396	0.483	0.579	0.414	0.925	0.432	0.638	0.635	0.641	0.966	0.595
DeepGO (selected)	**0.435**	0.444	0.426	0.896	0.399	0.503	0.577	0.447	0.928	0.438	**0.639**	0.643	0.635	0.967	0.592

*Note*: The DeepGOSeq model uses only sequence information. DeepGOFlat uses both the protein sequence and network interactions as input, but instead of hierarchically structured classification layers DeepGOFlat has one fully connected layer with sigmoid activation function to generate output predictions. Our final DeepGO model uses sequence and interaction networks with hierarchical classification layers. The first part of the evaluation shows performance results when considering all GO annotations (even those that our model cannot predict), while the second part focuses on the selected terms for which our model can generate predictions. Best performing models are highlighted in bold.

### 3.2 Incorporating protein networks

The majority of functions and biological processes in GO require multiple proteins to be performed. One source of information for proteins acting together can be obtained from protein–protein interaction networks. By adding information about protein–protein interactions, we planned to improve our model’s performance, in particular for prediction of associations to biological processes which usually require more than one protein to be performed. We encode protein–protein interactions as a multi-species knowledge graph of interacting proteins in which proteins within a species are linked through *interacts-with* edges and proteins in different species through a *orthologous-to* edge. We then apply a method to generate knowledge graph embeddings (Alshahrani *et al.*, 2017) to this graph and generate a vector representation for each protein. Furthermore, we integrate this vector representation with the protein sequence representation in our model, resulting in a multi-modal model that utilizes both protein sequences and protein interactions. Incorporating this network information significantly improves the performance for almost all GO classes, and the overall performance of our DeepGO method improves significantly in comparison with DeepGOSeq which uses only protein sequence as a feature, and in comparison to the BLAST baseline. Table 1 summarizes the results.

We find that the predictive performance of our model varies significantly between proteins in different organisms, in particular between single-cell and multi-cellular organisms. Table 2 summarizes the performance we achieve for individual organisms, and further broadly distinguishes between eukaryotic and prokaryotic organisms. We find that DeepGO achieves high performance for well-characterized model organisms, likely due to the rich characterization of protein functions in these organisms; other organisms do not have a large set of manually asserted function annotations and are therefore represented more sparsely in our evaluation set.

**Table 2.** Performance of our method distinguished by organisms

Organism	BP					MF					CC				
	F_max_	AvgPr	AvgRc	AUC	MCC	F_max_	AvgPr	AvgRc	AUC	MCC	F_max_	AvgPr	AvgRc	AUC	MCC
**Eukaryotes**	0.40	0.41	0.39	0.89	0.40	0.48	0.59	0.41	0.93	0.45	0.63	0.64	0.62	0.96	0.59
Human	0.42	0.46	0.39	0.89	0.42	0.51	0.64	0.42	0.94	0.46	0.60	0.58	0.61	0.96	0.56
Mouse	0.39	0.42	0.36	0.88	0.40	0.51	0.60	0.45	0.95	0.48	0.59	0.69	0.51	0.95	0.55
Rat	0.38	0.39	0.37	0.88	0.37	0.52	0.61	0.45	0.94	0.49	0.53	0.50	0.58	0.94	0.48
Fruit Fly	0.38	0.41	0.35	0.89	0.40	0.51	0.63	0.42	0.94	0.48	0.57	0.54	0.59	0.96	0.56
Yeast	0.45	0.46	0.43	0.93	0.46	0.42	0.49	0.37	0.91	0.38	0.57	0.55	0.59	0.96	0.56
Fission Yeast	0.42	0.43	0.41	0.91	0.41	0.40	0.40	0.39	0.91	0.35	0.77	0.77	0.78	0.98	0.74
Zebrafish	0.40	0.44	0.37	0.90	0.38	0.60	0.74	0.51	0.95	0.55	0.65	0.74	0.59	0.97	0.66
**Prokaryotes**	0.37	0.40	0.34	0.90	0.38	0.39	0.45	0.34	0.90	0.36	0.69	0.71	0.67	0.98	0.62
*E.coli*	0.40	0.42	0.38	0.93	0.42	0.40	0.47	0.35	0.93	0.38	0.73	0.76	0.70	0.99	0.66
Mycobacterium tuber-s	0.29	0.28	0.31	0.88	0.24	0.38	0.45	0.33	0.91	0.35	0.68	0.65	0.71	0.99	0.63
*Pseudomonas aeruginosa*	0.52	0.57	0.47	0.93	0.55	0.42	0.65	0.31	0.91	0.41	1.00	1.00	1.00	1.00	1.00
*Bacillus subtilis*	0.36	0.50	0.29	0.87	0.34	0.39	0.43	0.36	0.91	0.33	0.50	0.64	0.42	0.97	0.53

*Note*: We use the DeepGO model that combines both sequence and network information for this prediction. Best performance values are highlighted in bold.

We compare DeepGO with two top-performing methods in previous CAFA challenges (Radivojac *et al.*, 2013), FFPred3 (Cozzetto *et al.*, 2016) and GoFDR (Gong *et al.*, 2016), on a benchmark released as part of the CAFA3 competition. Neither DeepGO nor FFPred3 or GoFDR have used the protein annotations in this benchmark during training. Table 3 shows the performance results of DeepGO in comparison to FFPred3 and GoFDR on this benchmark set. DeepGO achieves the highest AUC in all three GO branches, while both FFPred3 and GoFDR outperform DeepGO in some GO branches on *F*_max_, precision, recall, or MCC.

**Table 3.** Evaluation of DeepGO, FFPred3 and GoFDR methods on a CAFA3 preliminary evaluation set

Method	BP					MF					CC				
	F _max_	AvgPr	AvgRc	AUC	MCC	F_max_	AvgPr	AvgRc	AUC	MCC	F_max_	AvgPr	AvgRc	AUC	MCC
FFPred3	0.26	0.30	0.23	0.83	0.23	0.38	0.35	**0.40**	0.86	0.29	0.44	0.46	0.43	0.89	0.39
GoFDR	0.20	0.27	0.15	0.61	0.00	**0.52**	**0.89**	0.36	0.84	**0.60**	0.40	0.40	0.41	0.72	0.31
DeepGO	**0.34**	**0.31**	**0.37**	**0.88**	**0.32**	0.47	0.61	0.39	**0.90**	0.37	**0.52**	**0.55**	**0.49**	**0.95**	**0.50**

The UniProt database may contain orthologous proteins which are almost identical and will have similar or identical functions. To ensure that our testing dataset does not contain sequences that are highly similar to sequences in our training dataset, we clustered the protein sequences by their sequence similarity. We computed pairwise sequence identity using BLAST (Altschul *et al.*, 1997) for all the proteins with experimental annotations. Then we clustered the protein sequences into two clusters by placing the sequences with at least 50% sequence identity in the first cluster and all other sequences in the second cluster. We used the first cluster as a training set and the second cluster as a testing set (both files are provided as Supplementary files S1 and S2). Our training set contains 45 342 sequences and our testing set contains 15 368 sequences. Table 4 show the performance of our model in the scenario where we evaluate on a set of sequences that are dissimilar to the sequences used in training.

**Table 4.** Evaluation of DeepGO on a dataset split by sequence identity

Model	*F*_max_	AvgPr	AvgRc	AUC	MCC
BP	0.397	0.437	0.364	0.900	0.395
MF	0.403	0.495	0.339	0.908	0.359
CC	0.625	0.654	0.598	0.963	0.598

We further evaluated how well DeepGO performs on different types of proteins. InterPro classifies proteins into families, domains and important sites (Finn *et al.*, 2017). We evaluate DeepGO’s performance by grouping proteins by their InterPro annotations. Supplementary Table S2 shows the performance for InterPro classes with at least 50 protein annotations in our test set. We find that for some important protein families, such as p53-like transcription factors (IPR008967), DeepGO can achieve high performance in all three GO ontologies, while for other kinds of proteins, such as those with a Ubiquitin-related domain (IPR029071), DeepGO fails to predict annotations to BP and MF accurately.

**Fig. 2.**
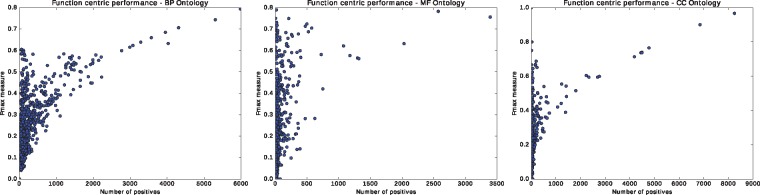
Term centric performance. These plots show the performance of our model for each term in our subset of GO as a function of the number of supporting proteins in test set which are annotated by the term

Using a term-centric evaluation measure (Radivojac *et al.*, 2013), we test how accurate our predictions are for different GO functions. Supplementary Table S3 shows the best performing GO functions from each ontology. Unsurprisingly, high-level functions with a large number of annotations generally perform significantly better than more specific functions. We further test whether the variance in predictive performance is intrinsic to our method or the result of different amounts of training data available for proteins of different families, with different domains, or for GO functions with different number of annotations. We plot the predictive performance of DeepGO as a function of the number of training samples in Figure 2, and observe that performance is strongly correlated with the number of training instances. However, due to the hierarchical nature of GO, an increased number of training instances will always be available for more general, high-level functions. In the future, additional weights based on information content of GO classes (Resnik, 1999) should be assigned to more specific functions which contain more information (Clark and Radivojac, 2013; Radivojac *et al.*, 2013); using these weights during training of our model may improve performance for more specific functions.

For automated annotation of a large number of proteins, such as the complete proteome of a newly sequenced organism, prediction time is also important. To determine the time needed for predicting functions and cellular locations of multiple proteins, we randomly selected 10 000 proteins of varying size from different organisms and performed function prediction with DeepGO using all three GO hierarchies, using an Intel Xeon E5-2680 CPU and an Nvidia GeForce GTX TITAN Z GPU. DeepGO requires 15GB of memory. As DeepGO relies on BLAST to identify a network embedding for a query protein, the majority of time (16 000 s, or 1.6 s per protein on average) was needed to perform the BLAST search. Actual prediction time for the neural network ranges between 2.2 ms per protein (for the CC model) to 3.5 ms per protein (for the BP model). Our results are similar to the reported results of GoFDR (Gong *et al.*, 2016) where the majority of time is required for BLAST search while actual prediction time is significantly faster.

## 4 Discussion

### 4.1 Multi-modal function prediction

Computational approaches to function prediction have been developed for many years (Radivojac *et al.*, 2013). One of the most basic approaches for function prediction has been the use of BLAST (Altschul *et al.*, 1997) to identify proteins with high sequence similarity and known functions, and assign the functions of the best matching protein to the protein to be characterized. Approaches for orthology-based function prediction include more comprehensive modelling of evolutionary relations, including relations between protein subdomains (Gaudet *et al.*, 2011), and these can outperform simple BLAST baseline experiments. Other approaches for function prediction rely on structure prediction. It is well known that protein ternary structure strongly influences a protein’s functions, but prediction of protein structure remains a challenging computational problem (Moult *et al.*, 2014), and even with known protein structure, functions cannot always be predicted accurately. Additionally, high-level physiological functions, such as *vocalization behavior* (GO: 0071625), will not be predictable from a single protein’s sequence or structure alone but require complex pathways and interacting proteins, all of which contribute to the function. For this purpose, several methods use protein–protein interaction networks to identify significant links between proteins that can be used to transfer functions, or significant network patterns that may be predictive of a function (Baryshnikova, 2016; Jiang and McQuay, 2012; Kirac and Ozsoyoglu, 2008; Nguyen *et al.*, 2011).

While many of these approaches rely on hand-crafted features, some approaches already applied feature learning (i.e. deep learning) to parts of these data types. For example, feature learning approaches have significantly improved the prediction of transcription factor binding sites and functional impact of genomic variants (Alipanahi *et al.*, 2015; Zhou and Troyanskaya, 2015), and DeepGO also utilizes feature learning on protein–protein interaction networks (Alshahrani *et al.*, 2017). Here, we have extended the application of deep learning approaches in function prediction in three ways: first, we apply feature learning through the use of a CNN and embedding layer to learn a representation of protein sequence; second, we developed a deep, ontology-structured classification model that can refine features on each distinction present in the GO; and third, we use multi-modal data sources, in particular the protein sequence and information from protein–protein interaction networks, within a single model. Through the multi-modal nature of our machine learning model, other types of data can be integrated within the DeepGO model as long as they can be used as input to a representation learning method that learns vector representations. For example, protein structure information, if available, could be incorporated in our model by adding another feature learning branch that generates dense, low-dimensional representations of protein structure (Wang *et al.*, 2017) and using these as input to our hierarchical classifier. Furthermore, established function prediction methods use several additional sources of information to generate features for function prediction, including co-expression (Wass *et al.*, 2012), classification in functional protein families based on protein domains (Das *et al.*, 2015), and phylogenetic information (Engelhardt *et al.*, 2011). Adding these additional sources of information may help to further improve DeepGO’s performance in the future.

### 4.2 Hierarchical classification on ontologies

In addition to the multi-modal nature of features used in DeepGO, another contribution of our work is the deep hierarchical classification model that optimizes predictive performance on whole hierarchies, accounts for class dependencies (i.e. the semantics of annotations in GO) during training time, learns features in a hierarchical manner, and is optimized jointly together with the feature learning component of our model in an end-to-end manner. Our method can be applied to other applications with a similarly structured output space and which rely on learning feature representations. In particular, we plan to apply our model for predicting disease associations of genes which are encoded using the Disease Ontology (Osborne *et al.*, 2009), or phenotype associations of genetic variants which are encoded using phenotype ontologies (Gkoutos *et al.*, 2017).

The advantages of our model are its potential for end-to-end learning, the global optimization and the potential to predict any class given sufficient training data. In particular the end-to-end learning provides benefits over approaches such as structured support vector machines (Sokolov and Ben-Hur, 2010), which generally rely on hand-crafted feature vectors.

However, our model also has disadvantages. First, it needs large amounts of training data for each class; this data is readily available through the manual GO annotations that have been created for many years, but will not easily be available for other areas of application, such as predicting phenotype annotations or effects of variants. Furthermore, our model is complex and requires large computational resources for training, and therefore may not be applicable in all settings.

In the future, we intend to extend our hierarchical model in several directions. First, we plan to include more information from GO, in particular parthood relations and regulatory relations, which may provide additional information. We will also explore adding more features, such as additional types of interactions (genetic interactions, or co-expression networks), and information extracted from text.

## Supplementary Material

Supplementary Data
