# Supplementary material for: DeepGO: predicting protein functions from sequence and interactions using a deep ontology-aware classifier
Source: Bioinformatics. 2017 Oct 3;34(4):660–8. doi: 10.1093/bioinformatics/btx624 (PMC5860606; doi:10.1093/bioinformatics/btx624)
Supplement: Supplementary Data [file btx624_supp.zip › btx624-suppl_data/supplement.pdf]

Supplementary materials: DeepGO: Predicting protein functions from sequence and interactions using a deep ontology-aware classifier

Embedding size	Filter number	BP val_loss	MF val_loss	CC val_loss
32	16	0.09797	0.04559	0.06038
32	32	0.09728	0.04536	0.06036
32	64	0.09681	0.04568	0.06055
32	128	0.09697	0.04513	0.06007
64	16	0.09698	0.04570	0.06097
64	32	0.09701	0.04599	0.06014
64	64	0.09767	0.04542	0.05997
64	128	0.09725	0.04563	0.05939
128	16	0.09720	0.04609	0.06085
128	32	0.09744	0.04606	0.06024
128	64	0.09684	0.04569	0.05991
128	128	0.09809	0.04583	0.05967
256	16	0.09790	0.04576	0.06066
256	32	0.09735	0.04589	0.06027
256	64	0.09759	0.04561	0.05957
256	128	0.09706	0.04594	0.05862
512	16	0.09855	0.04610	0.06138
512	32	0.09700	0.04686	0.06013
512	64	0.09682	0.04719	0.05974
512	128	0.09846	0.04594	0.05911

Table 1: Validation losses of the models for different embedding size and number of convolution filters.

InterPro	InterPro Name	BP			MF			CC		
		F_{max}	Avg Pr	Avg Rc	F_{max}	Avg Pr	Avg Rc	F_{max}	Avg Pr	Avg Rc
IPR029063	S-adenosyl-L-methionine-dependent methyltransferase	0.49	0.49	0.49	0.43	0.54	0.36	0.74	0.71	0.77
IPR013087	Zinc finger C2H2-type	0.49	0.44	0.55	0.56	0.48	0.67	0.76	0.72	0.81
IPR008967	p53-like transcription factor, DNA-binding	0.48	0.49	0.48	0.62	0.57	0.67	0.82	0.79	0.85
IPR000504	RNA recognition motif domain	0.47	0.46	0.49	0.72	0.72	0.73	0.67	0.62	0.73
IPR023313	Ubiquitin-conjugating enzyme, active site	0.46	0.40	0.53	0.63	0.60	0.66	0.67	0.63	0.73
IPR019775	WD40 repeat, conserved site	0.46	0.42	0.50	0.53	0.55	0.51	0.69	0.68	0.70
IPR009071	High mobility group box domain	0.46	0.46	0.45	0.64	0.67	0.60	0.74	0.71	0.78
IPR001680	WD40 repeat	0.45	0.44	0.47	0.51	0.55	0.48	0.68	0.67	0.68
IPR015943	WD40/YVTN repeat-like-containing domain	0.45	0.44	0.46	0.52	0.64	0.44	0.66	0.65	0.66
IPR016135	Ubiquitin-conjugating enzyme/RWD-like	0.45	0.40	0.51	0.61	0.61	0.62	0.67	0.64	0.72
IPR011991	Winged helix-turn-helix DNA-binding domain	0.44	0.45	0.44	0.43	0.55	0.35	0.64	0.62	0.66
IPR009057	Homeobox domain-like	0.44	0.44	0.44	0.61	0.64	0.59	0.74	0.71	0.78
IPR017441	Protein kinase, ATP binding site	0.43	0.41	0.45	0.63	0.63	0.64	0.58	0.55	0.60
IPR000727	Target SNARE coiled-coil homology domain	0.43	0.51	0.37	0.57	0.78	0.45	0.56	0.72	0.46
IPR008271	Serine/threonine-protein kinase, active site	0.43	0.40	0.45	0.63	0.65	0.61	0.59	0.57	0.61
IPR011009	Protein kinase-like domain	0.41	0.40	0.43	0.60	0.60	0.60	0.59	0.57	0.61
IPR013083	Zinc finger, RING/FYVE/PHD-type	0.40	0.40	0.41	0.48	0.54	0.43	0.66	0.63	0.70
IPR016040	NAD(P)-binding domain	0.40	0.43	0.38	0.35	0.52	0.26	0.72	0.69	0.75
IPR000980	SH2 domain	0.40	0.36	0.43	0.51	0.58	0.45	0.68	0.63	0.74
IPR011993	PH domain-like	0.39	0.42	0.37	0.45	0.60	0.35	0.56	0.54	0.58
IPR020846	Major facilitator superfamily domain	0.39	0.36	0.43	0.54	0.57	0.52	0.61	0.65	0.57
IPR028889	Ubiquitin specific protease domain	0.39	0.35	0.43	0.27	0.41	0.20	0.66	0.63	0.69
IPR018200	Ubiquitin specific protease, conserved site	0.39	0.35	0.43	0.27	0.41	0.20	0.65	0.63	0.68
IPR001452	SH3 domain	0.38	0.39	0.37	0.45	0.57	0.37	0.56	0.57	0.56
IPR011992	EF-hand domain pair	0.37	0.43	0.33	0.50	0.52	0.48	0.66	0.69	0.63
IPR027417	P-loop containing nucleoside triphosphate hydrolase	0.37	0.40	0.35	0.42	0.64	0.32	0.63	0.65	0.62
IPR029071	Ubiquitin-related domain	0.35	0.34	0.37	0.42	0.46	0.38	0.63	0.62	0.64
IPR000008	C2 domain	0.35	0.41	0.30	0.47	0.73	0.35	0.49	0.62	0.40
IPR032675	Leucine-rich repeat domain, L domain-like	0.34	0.43	0.28	0.49	0.64	0.39	0.45	0.49	0.41
IPR012336	Thioredoxin-like fold	0.33	0.36	0.31	0.47	0.50	0.45	0.69	0.69	0.69
IPR013783	Immunoglobulin-like fold	0.33	0.38	0.29	0.52	0.64	0.43	0.49	0.51	0.47
IPR017907	Zinc finger, RING-type, conserved site	0.31	0.38	0.27	0.38	0.63	0.27	0.56	0.50	0.64
IPR013320	Concanavalin A-like lectin/glucanase domain	0.29	0.36	0.25	0.34	0.52	0.25	0.55	0.51	0.59

Table 2: Performance of DeepGO by InterPro domains. Only InterPro domains for which at least 50 proteins are in our evaluation dataset are included in this evaluation.

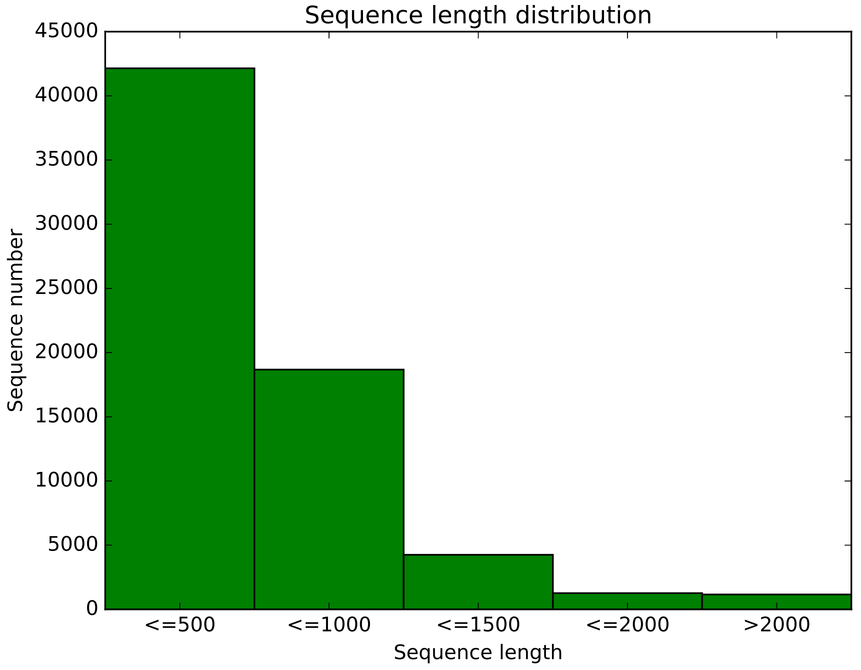

Figure 1: Sequence length distribution of SwissProt proteins with experimental annotations

Function	Label	DeepGO		DeepGOSeq	
		F_{max}	ROC AUC	F_{max}	ROC AUC
**Biological Process**					
GO:0009987	cellular process	0.794114	0.664092	0.793314	0.540904
GO:0044699	single-organism process	0.743470	0.681554	0.738722	0.572571
GO:0065007	biological regulation	0.706243	0.744459	0.676651	0.684731
GO:0008152	metabolic process	0.639053	0.747201	0.542575	0.603799
GO:0032502	developmental process	0.539157	0.686034	0.383134	0.613468
GO:0051179	localization	0.499622	0.656205	0.321163	0.570820
GO:0050896	response to stimulus	0.475592	0.699616	0.393251	0.521504
GO:0071840	cellular component organization or biogenesis	0.448219	0.692364	0.359989	0.564579
GO:0032501	multicellular organismal process	0.393939	0.553874	0.219520	0.552407
GO:0022414	reproductive process	0.355517	0.532847	0.184811	0.544875
GO:0040007	growth	0.298422	0.232787	0.073167	0.448431
GO:0002376	immune system process	0.289439	0.188714	0.086750	0.459047
GO:0051704	multi-organism process	0.283341	0.499442	0.163127	0.535393
GO:0040011	locomotion	0.216931	0.235413	0.083333	0.296802
GO:0007610	behavior	0.198529	0.155232	0.050633	0.546377
GO:0023052	signaling	0.190000	0.114028	0.027231	0.195022
GO:0022610	biological adhesion	0.170940	0.111006	0.065306	0.216923
GO:0048511	rhythmic process	0.125000	0.032131	0.020725	0.093639
GO:0000003	reproduction	0.047312	0.014864	0.012214	0.002375
**Molecular Function**					
GO:0003824	catalytic activity	0.782625	0.785353	0.696710	0.697225
GO:0005488	binding	0.756233	0.785914	0.729583	0.727175
GO:0005215	transporter activity	0.704724	0.228738	0.584327	0.333024
GO:0001071	nucleic acid binding transcription factor activity	0.567164	0.311635	0.362490	0.356181
GO:0060089	molecular transducer activity	0.529915	0.204974	0.466346	0.244261
GO:0004871	signal transducer activity	0.528226	0.296622	0.431461	0.474962
GO:0098772	molecular function regulator	0.359736	0.277201	0.203125	0.567162
GO:0016209	antioxidant activity	0.278788	0.017432	0.075472	0.023356
GO:0000988	transcription factor activity, protein binding	0.263959	0.157683	0.150476	0.267753
GO:0005198	structural molecule activity	0.240437	0.190938	0.045262	0.317486
GO:0009055	electron carrier activity	0.193548	0.017501	0.012821	0.018585
GO:0045182	translation regulator activity	0.033333	0.008364	0.018018	0.000856
**Cellular Component**					
GO:0044464	cell part	0.966916	0.835707	0.966806	0.685692
GO:0043226	organelle	0.765235	0.634356	0.711517	0.647867
GO:0044422	organelle part	0.598980	0.651945	0.493126	0.625321
GO:0016020	membrane	0.594333	0.717554	0.493558	0.706837
GO:0032991	macromolecular complex	0.481339	0.685256	0.335529	0.643006
GO:0044421	extracellular region part	0.480000	0.288240	0.134766	0.533930
GO:0005576	extracellular region	0.442006	0.295835	0.374728	0.679528
GO:0044425	membrane part	0.420295	0.520572	0.296134	0.661042
GO:0044456	synapse part	0.373494	0.058588	0.020293	0.039199
GO:0045202	synapse	0.352941	0.027405	0.009554	0.001589
GO:0031974	membrane-enclosed lumen	0.257282	0.184263	0.038957	0.415858
GO:0099512	supramolecular fiber	0.253521	0.026227	0.057143	0.024539
GO:0030054	cell junction	0.232558	0.213859	0.055427	0.482607
GO:0031012	extracellular matrix	0.200000	0.054635	0.020619	0.023618
GO:0044420	extracellular matrix component	0.190476	0.001616	0.000000	-0.000000
GO:0005623	cell	0.133333	0.007377	0.000000	-0.000000
GO:0044217	other organism part	0.085517	0.034413	0.017241	0.003086
GO:0009295	nucleoid	0.077922	0.004509	0.021505	0.000185
GO:0019012	virion	0.071429	0.028803	0.000000	-0.000000
GO:0044423	virion part	0.062112	0.027729	0.022472	0.000109

Table 3: Performance of DeepGO distinguished by GO functions.
